# SARS-CoV-2 Antigenemia as a Confounding Factor in Immunodiagnostic Assays: A Case Study

**DOI:** 10.3390/v13061143

**Published:** 2021-06-14

**Authors:** Konstantinos Belogiannis, Venetia A. Florou, Paraskevi C. Fragkou, Stefanos Ferous, Loukas Chatzis, Aikaterini Polyzou, Nefeli Lagopati, Demetrios Vassilakos, Christos Kittas, Athanasios G. Tzioufas, Sotirios Tsiodras, George Sourvinos, Vassilis G. Gorgoulis

**Affiliations:** 1 Molecular Carcinogenesis Group, Department of Histology and Embryology, Medical School, National and Kapodistrian University of Athens, GR-11527 Athens, Greece; k.belojohn91@outlook.com (K.B.); venflorou@hotmail.com (V.A.F.); apolyzou@med.uoa.gr (A.P.); nefeli.lagopati@gmail.com (N.L.); dvassilakos@gmail.com (D.V.); ckittas@med.uoa.gr (C.K.); 2 4th Department of Internal Medicine, Attikon University Hospital, National and Kapodistrian University of Athens, GR-12462 Athens, Greece; evita.fragou@gmail.com (P.C.F.); sotirios.tsiodras@gmail.com (S.T.); 3 2nd Medical Department, General Hospital of Athens G. Gennimatas, GR-11527 Athens, Greece; stefanos.ferous@hotmail.com; 4 Department of Pathophysiology, Athens School of Medicine, National and Kapodistrian University of Athens, GR-11527 Athens, Greece; lukechatzis@gmail.com (L.C.); agtzi@med.uoa.gr (A.G.T.); 5 Manchester Academic Health Sciences Centre, Division of Cancer Sciences, University of Manchester, Manchester M13 9NQ, UK; 6 Laboratory of Clinical Virology, Medical School, University of Crete, Crete, GR-71003 Heraklion, Greece; 7 Biomedical Research Foundation, Academy of Athens, GR-11527 Athens, Greece; 8 Center for New Biotechnologies and Precision Medicine, Medical School, National and Kapodistrian University of Athens, GR-11527 Athens, Greece

**Keywords:** SARS-CoV-2, COVID-19, viremia, antigenemia, antibody, seroconversion, immunoprevalence, non-responders, ELISA, immunodiagnostics

## Abstract

Humoral immunity has emerged as a vital immune component against severe acute respiratory syndrome coronavirus 2 (SARS-CoV-2). Nevertheless, a subset of recovered Coronavirus Disease-2019 (COVID-19) paucisymptomatic/asymptomatic individuals do not generate an antibody response, constituting a paradox. We assumed that immunodiagnostic assays may operate under a competitive format within the context of antigenemia, potentially explaining this phenomenon. We present a case where persistent antigenemia/viremia was documented for at least 73 days post-symptom onset using ‘in-house’ methodology, and as it progressively declined, seroconversion took place late, around day 55, supporting our hypothesis. Thus, prolonged SARS-CoV-2 antigenemia/viremia could mask humoral responses, rendering, in certain cases, the phenomenon of ‘non-responders’ a misnomer.

## 1. Background

Severe acute respiratory syndrome coronavirus 2 (SARS-CoV-2), the culprit of an ongoing pandemic, continues to engender detrimental effects on healthcare systems worldwide leading to serious socioeconomic consequences. Following an incubation period of 2-14 days infected individuals experience a heterogeneous clinical course of the so-called Coronavirus Disease-2019 (COVID-19), ranging from asymptomatic infection to critical illness [1,2]. Similarly, symptomatic infection comprises of a wide array of clinical manifestations from localized disease affecting preferentially the respiratory and occasionally the gastrointestinal tract to multisystemic organ involvement [3,4].

The brisk induction of proinflammatory responses, the so-called cytokine storm syndrome, is currently regarded as the major contributor of COVID-19-related multiorgan dysfunction [5]. However, there is sparse evidence that contradicts this supposition, implying a possible underestimated degree of viral-induced organ cytotoxicity [6]. Although the route of viral dissemination to other organs is still a subject of debate, growing evidence suggests this occurs hematogenously [4,5,7]. Nonetheless, SARS-CoV-2 viremia and antigenemia have only been documented in disproportionally lower rates than expected and the clinical significance of these parameters remains undetermined [8,9,10,11].

**Figure 1:**
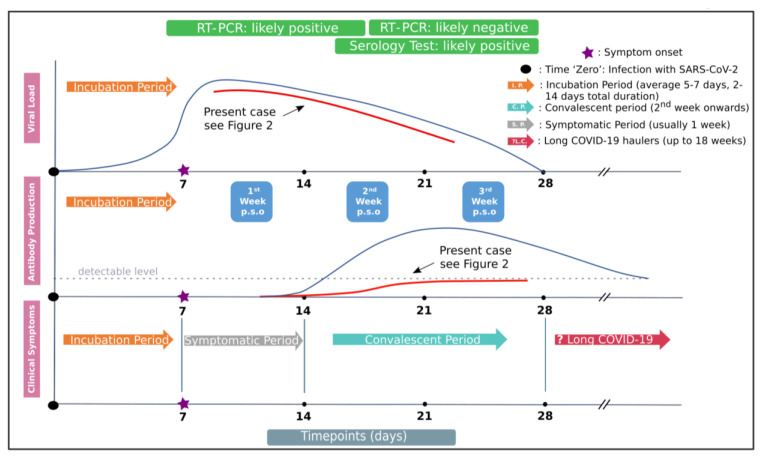
Diagram summarizing antibody responses, viral load (obtained from upper respiratory specimens) and associated clinical course. Antibody responses and viral load temporal kinetics as they correlate with clinical symptoms are depicted. Estimated time intervals are based on data from several published studies. The line graph in red qualitatively illustrates the viral load in nasopharyngeal swabs and the antibody response pattern of our patient—who is considered as late/non-responder—relatively to that mounted in most COVID-19 individuals.

Besides the role of antigenemia/viremia in COVID-19 pathogenesis, another variable aspect of the disease is the host’s immune response against SARS-CoV-2, and more specifically, the diversity of the humoral response level among SARS-CoV-2-infected patients. Based on currently available serological assays, it is evident that the majority of COVID-19 patients seroconvert within 2 weeks post symptom onset (p.s.o), whereas delayed (beyond the 2nd week p.s.o) or even absent antibody responses (non-responders) have also been documented (Figure 1) [12]. The latter is particularly true in asymptomatic or paucisymptomatic patients [12]. This divergent pattern of humoral response has also been observed in the other two beta-coronaviruses (SARS-CoV and Middle East respiratory syndrome coronavirus, MERS-CoV) and other viral strains, such as human papillomavirus (HPV) and human rhinoviruses [12,13,14]. This phenomenon of undetectable antibody titers following convalescence constitutes a paradox, which has neither been studied nor satisfactorily explained.

To explain this paradox in COVID-19, we sought to examine the hypothesis that the presence of viral antigens in serum (antigenemia) could mask seroconversion by their binding to circulating antibodies. In such a competitive environment, antibody detection may be compromised in immunoassays. If this scenario is valid, then two conditions should be met: (1) viral antigen(s) should be detected in the blood stream and (2) whether the absence of seroconversion is due to the presence of antigen–antibody complexes, then progressive decrease of antigenemia should be followed by increasingly detectable antibody levels. Herein, we report, to the best of our knowledge, evidence as proof-of-concept supporting the proposed supposition.

A 19-year-old male presented on 12 October 2020 (day 1) with a 24-h history of fever up to 38.4 °C, without any additional signs or symptoms (Table 1). His past medical history was significant for prediabetes treated with metformin while no history of primary or secondary immunodeficiency was reported. Due to a recent history of close contact with a confirmed COVID-19 case, a nasopharyngeal swab was obtained and tested for SARS-CoV-2 with reverse transcription polymerase chain reaction (RT-PCR) on 13 October 2020 (day 2) p.s.o, confirming the diagnosis (cycle threshold value (Ct) = 15). Subsequently, the patient self-isolated at home for two weeks, as per national infection control protocols. His fever subsided within a few days. Besides antipyretics, the patient did not receive any other medications. However, upon completion of the two-weeks’ isolation, low grade fever (up to 37.4 °C) recurred. A repeat RT-PCR test performed on a nasopharyngeal swab on 27 October 2020 (day 16) p.s.o. was positive (Ct = 30) (Table 1). Fever finally resolved on 29 October, which is 18 days after symptom onset.

A series of serum samples for the detection of antibodies against SARS-CoV-2 were collected at regular intervals (every 4–5 days) starting on day 6 p.s.o up to day 27 p.s.o, followed by two additional samples on days 55 and 73 (Table 1), a timeframe that exceeded the expected seroconversion window (Figure 1).

## 2. Methods

### 2.1. Serum Isolation and Handling

Whole blood from patients was collected in the vacuumed gel separator and clot activator tubes (reference number: 44718, FL Medical) and then centrifuged at 2000 rpm for 8 min. Shortly after centrifugation, sera were loaded onto freshly made ELISA plates for antigen and antibody detection as described in more detail below. Upon usage, the remaining serum was transferred to a sterile cryovial for long cryopreservation at −80 °C. Sera unable to be tested within the same day of collection were similarly stored at −80 °C prior to their analysis. In case of hemolysis, sera were discarded and excluded in all experimental steps involved in the development of our ‘in-house’ assays.

### 2.2. Serum Characteristics

All sera used in the present study were used shortly after isolation or within the same day when this was not possible. Hemolytic, icteric, and lipidemic sera were not used as it has been shown that they may result in non-specific interferences in ELISA [15]. Pre-COVID-19 sera used as negative controls for the development of our ‘in-house’ assays were stored at −80 °C and used for testing after one freeze–thaw cycle. Plasma samples were not used during assay development or testing in the present study.

### 2.3. Monoclonal Antibody Production

The monoclonal antibodies 480-S2 and 479-G2 used in the present immunoassays have been generated by immunizing mice against the receptor binding domain (RBD) region of the spike (S) protein of SARS-CoV-2 via a modification of the method described by Koehler and Milstein [16]. Following vigorous immunosorbent assay selection cycles, clones exhibiting the required sensitivity, specificity and reproducibility were selected for downstream applications as detailed below. These have been extensively validated (Supplementary Figure S1A) and are under proprietary rights (patent application no.:22-0003846810). The validation process was undertaken on three distinct settings (archival material, nasopharyngeal swabs, and serum) (Supplementary Figure S1) using appropriate positive and negative controls, as detailed in the corresponding sections below.

### 2.4. Double Antigen ELISA for Antibody Detection

For antibody detection in the patient’s serum, an ‘in-house’ double antigen ELISA detecting antibodies directed against the trimeric S protein (S-trimer, Trenzyme, GmBH, Germany) was developed as follows: high-binding plates precoated with 1.5 μg/mL S-trimer were blocked with 300 μL of 4% bovine serum albumin (BSA) following incubation for one hour at room temperature (RT). Then, 50 μL of serum samples were loaded in duplicates (1:1 dilution ratio) and incubated at 4 °C overnight. Next, 50 μL/well of S protein conjugated to horseradish peroxidase (S-HRP) (5:12,000 dilution) were loaded and incubated for 45 min at RT. After appropriate washing, 50 μL/well of 3,3′,5,5′-Tetramethylbenzidine (TMB) were added and let to incubate for ten minutes at RT in the dark. Subsequently, 50 μL of phosphoric acid were used for reaction termination and the absorption was quantified using a microplate reader at 450 nm (cut-off value: 0.100). Washing was performed at appropriate steps using phosphate buffer saline/0.1% Tween (PBSTx5).

Following testing on 150 negative pre-COVID-19 and 250 RT-PCR positive samples, validation data revealed 90.5% sensitivity and 95% specificity (Supplementary Figure S1(Bi)). Number of freeze–thaw cycles and different cryopreservation time periods of sera does not affect methodology. The same assay was also cross-referenced to an FDA-approved enzyme linked immunosorbent assay (ELISA) (Euroimmun, Luebeck, Germany), by testing a panel of 321 anonymized samples (Pearson’s Chi-squared test with Yates’ continuity correction; *p*-value = 0.5288) [17]. Additionally, a commercially available rapid antibody detection test (ProGnosis Biotech, Catalog number: V1210/V1230) was also implemented for comparison purposes.

### 2.5. Sandwich ELISA for SARS-CoV-2 Antigen Detection

For SARS-CoV-2 antigen detection in patient’s serum, a sandwich ‘in-house’ ELISA assay was developed as follows: high-binding plates precoated with 2 μg/mL of monoclonal antibody 480-S2 were blocked with 300 μL of 4%BSA/0.05%Tween blocking buffer following incubation for one hour at 37 °C. Subsequently, 50 μL of serum samples (1:1 dilution ratio) were loaded in duplicates and were allowed to incubate overnight at 4 °C. Following adequate washes with PBST, 50 μL of secondary antibody 479-G2 solution labelled with HRP (G2-HRP at a concentration of 1:50,000) were loaded into each well, and after a 30 min incubation period at RT in the dark, development of the signal was performed using 50 μL of TMB substrate. Following a ten-minute incubation time, equal volume of phosphoric acid was introduced to terminate the reaction and signal was quantified as above (cut-off value: 0.100). Performance characteristics of the test were calculated following testing on 100 samples (28 RT-PCR positive and 72 negative pre-COVID-19 samples), revealing a sensitivity of 93% and specificity of 99% (Supplementary Figure S1(Bii)). Number of freeze–thaw cycles and different cryopreservation time periods of sera does not affect methodology.

### 2.6. Immunohistochemistry

Immunohistochemistry was performed using the aforementioned anti-SARS-CoV-2 monoclonal antibody (479-G2). The Novolink Polymer Detection System (Leica Biosystems, Vamvakas, Athens, Greece) was used for development of the signal and hematoxylin for counterstaining (Supplementary Figure S1A). The specificity of the immunohistochemical signal was confirmed by (1) omitting the primary antibody and (2) performing competition with the corresponding S antigen (Supplementary Figure S1A). Tissues from a large cohort of non-COVID-19 patients served as negative controls, whereas viral particles were detected in archival material from lung tissues of COVID-19 patients [18,19].

### 2.7. RNA Extraction and Real-Time qPCR

RNA was extracted using the NucleoSpin Virus RNA purification kit (Macherey-Nagel #740.983, Lab Supplies, Athens, Greece) according to the manufacturer’s instructions and as previously described [20]. RT-qPCR was performed utilizing the One Step PrimeScript III RT-PCR Kit (Takara # RR601B, Lab Supplies, Athens, Greece) on a Rotor-Gene Q 6000 (Qiagen) thermal cycler following the manufacturer’s instructions and using the CDC N-gene (IDT, BioLine, Athens, Greece) directed primers (https://www.cdc.gov/coronavirus/2019-ncov/lab/rt-pcr-panel-primer-probes.html; accessed on: 20 May 2021).

### 2.8. Next Generation Sequencing

Next generation sequencing (NGS) of the viral genome was performed (Supplementary Figure S2) as follows: the Ion AmpliSeq Library Kit Plus was used to generate libraries following the manufacturer’s instruction, employing the Ion AmpliSeq SARS-CoV-2 RNA custom primers panel (ID: 05280253, Thermo Fisher Scientific, AntiSel, Athens, Greece). Briefly, library preparation steps involved reverse transcription of RNA using the SuperScript VILO cDNA synthesis kit (Thermo Fisher Scientific), 17–19 cycles of PCR amplification, adapter ligation, library purification using the AgencourtAMPure XP (Beckman Coulter, Leriva SA, Athens, Greece)and library quantification using Qubit Fluorometer high-sensitivity kit. Ion 530 Chips were prepared using Ion Chef and NGS reactions were run on an Ion GeneStudio S5, ion torrent sequencer (Thermo Fisher Scientific). Raw data (FASTA sequence) of isolated viral strain are available under the submission code EPI_ISL_856971 at GISAID Initiative (https://www.gisaid.org/; accessed on: 22 January 2021).

### 2.9. Bioinformatics

The SARS-CoV-2 Wuhan-Hu-1 strain complete genome was used as reference for alignment. Both, AmpliSeq alignments and quality controls were performed using the Torrent Server of Ion Torrent S5 sequencer employing default settings. Aligned reads served for both reference-guided assembly and variant calling. Assembly was performed using the iterative refinement meta-assembler (IRMA v0.6.1) that produced a consensus sequence (per sample) using a >50% cut-off for calling single nucleotide polymorphism.

### 2.10. Data and Statistical Analysis

Line graph representing the optical density (O.D) values of antigen and anti-SARS-CoV-2 antibody titers as a function of time was constructed with GraphPad Prism version 7.00 (www.graphpad.com; accessed on 15 May 2021). Statistically significant differences between the two methods ‘in-house’ ELISA and Euroimmun were evaluated by Pearson’s Chi-squared test with Yates’ continuity correction as appropriate. *p* < 0.05 was considered statistically significant. The samples were examined in duplicates.

### 2.11. Ethical Statement

Written informed consent was obtained by the patient for the collection and processing of the samples and for the publication of this case report. This study was conducted within the frame of ‘Emblematic action to handle SARS-CoV-2 infection: Epidemiological study in Greece via extensive testing for viral and antibody detection, sequencing of the virome and genetic analysis of the carriers’, which has been approved by the Ethics Committee of Medical School of National Kapodistrian University of Athens (Approval No. 317/12-06-2020). Finally, all animal experiments were performed in accordance with ethical standards of the responsible committee on human experimentation and with the Helsinki Declaration of 1975, as revised in 1983.

## 3. Results

**Figure 2:**
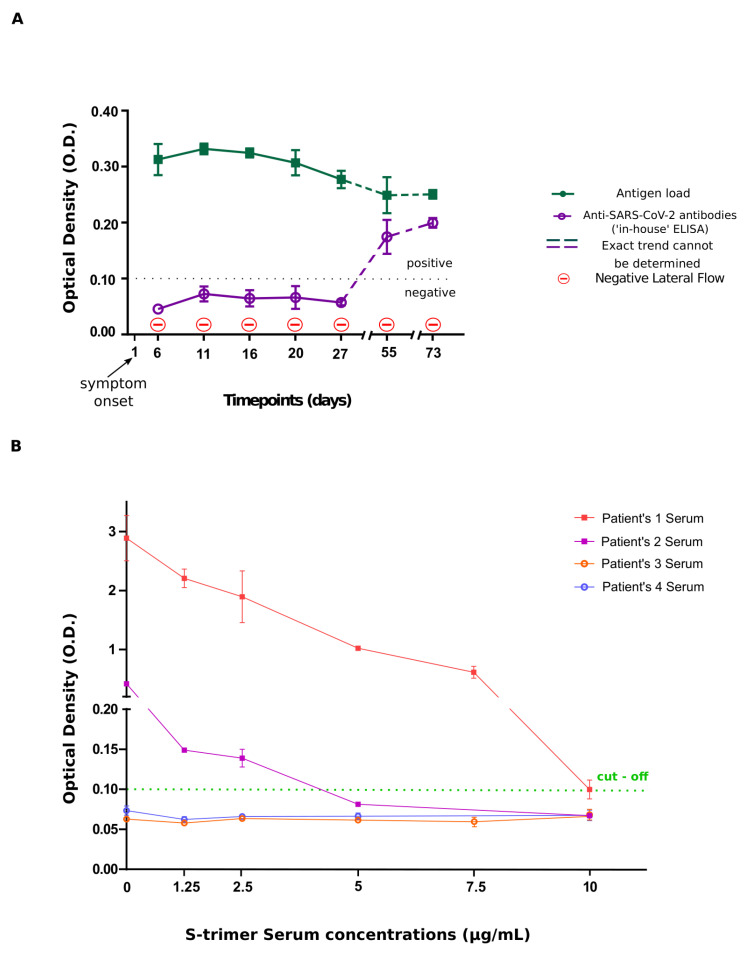
(**A**) Diagram depicting antigen load in parallel with antibody trends as a function of time and (**B**) experiment demonstrating antibody masking through exponentially increasing S-antigen concentrations. (**A**) Diagram depicting kinetics of antigen/viral load as relate to antibody generation detected by two separate immunodiagnostic assays (‘in-house’ ELISA and lateral flow). Time-dependent decline in the antigen load is associated with seroconversion 55-days post symptom onset in the case of our ‘in-house’ ELISA, whilst it failed to occur according to the lateral flow assay. (**B**) ELISA plate demonstrating OD readings at 450 nm of spiked known positive (high antibody titers, no antigen)—(patients 1 and 2) and negative (no antibody, no antigen)—(patients 3 and 4) control serum samples. Sequentially decreasing OD values accompanies the sequential increase in S-antigen concentration in the spiked positive control samples (patients 1 and 2). A transition from positive to negative result for the presence of antibodies takes place at an S-antigen concentration of 10 μg/mL and 5 μg/mL for patients 1 and 2, respectively.

Patient’s serum was examined concurrently for the presence of viral S-antigen and anti-SARS-CoV-2 immunoglobulins during the period of 73 days. During his disease course, serial serum samples were obtained and tested negative for the presence of antibodies within the first month p.s.o (expected seroconversion window). According to the first part of our hypothesis, we sought to examine the presence of antigenemia using our ‘in-house’ methodology. Interestingly, persistent viral S-antigen presence was observed for at least 73 days after the onset of symptoms, supporting existing evidence regarding antigen detection in the blood [11,21]. With regards to the second part of our assumption, monitoring of the kinetics of the two parameters under investigation showed a progressive decline of S-antigenemia that was accompanied by a gradual increase of antibody titers, resulting in evident seropositivity, beyond the expected seroconversion window (Figure 1 and Figure 2A). Notably, lateral flow testing failed to detect seroconversion (Figure 2A).

To functionally recapitulate the above observation (masking of humoral response by antigenemia), serum from two separate patients (patients 1 and 2 with history of confirmed COVID-19 diagnosis) with different levels of antibody and undetectable antigenemia titers, as per our suggested workflow, (positive controls) were spiked with increments of S-trimer protein diluted in PBS. S-trimer concentrations of 1.25 μg/mL, 2.5 μg/mL, 5 μg/mL, and 10 μg/mL were used (total volume 25 μL) along with two pre-COVID-19 sera, which served as negative controls (patients 3 and 4). Serum samples and S-trimer solution were added in a 1:1 volume ratio (50 μL total volume) and the resulting mixture was incubated for 1 h at 37 °C followed by overnight incubation at 4 °C to allow for antibody complexes to form. These spiked samples were loaded on our double antigen ELISA platform for antibody detection as described in methods section. In support to our hypothesis, the positive control samples spiked with 10 μg/mL and 5 μg/mL of S-trimer, respectively were confirmed as being negative with an OD value lower than or equal to the cut-off value (Figure 2B). This finding strengthens our observations and emphasizes the existence of a threshold ratio between antibody/antigens, above which antibody detection can occur with available assays.

Finally, to exclude presence of S protein-associated mutations that could affect antibody affinity and thus, potentially account for such results, we performed genome wide sequencing of the isolated strain. Sequencing revealed a novel strain showing 99% similarity with Wuhan-Hu-1 variant and bioinformatic analysis demonstrated presence of two S-related mutations, including the D614G and A879S (Supplementary Figure S2). As seroconversion did occur, it is unlikely that these mutations are able to affect the performance of available immunoassays.

## 4. Discussion

Immune responses against SARS-CoV-2 have challenged the scientific community worldwide, mainly due to their high interindividual variability [2]. Heightened immune responses have been associated with adverse clinical outcomes while non-detectable ones, characterize mainly asymptomatic and mild cases [12,22]. The absence of humoral immunity has been attributed to various factors including variability in disease severity and host-related characteristics [23]. With regards to the latter, augmented innate over adaptive immune responses and/or a robust T-cell-mediated viral clearance are proposed as possible explanations [24]. However, this notion was disputed by recent studies demonstrating presence of dominant B-cell over T-cell responses irrespective of disease severity along with longitudinal persistence in memory specific T- and B-cells in mild cases [25,26]. This emphasizes the importance of humoral immunity in viral clearance and points towards a different explanation, whilst raising an important question: does the phenomenon of non-responders reflect a true immunobiological event or a limitation of currently available diagnostic tools in precisely portraying the immune landscape?

According to a study comparing different antibody detecting assays, this observation was reproducible by all methods, implying that absence of seroconversion represents a biological rather than a technical issue [23]. To the contrary, serum of presumably non-responders inhibited cell line infection upon culture with viable SARS-CoV-2, as assessed by a neutralization assay, the gold standard for antibody efficiency [12,24]. The latter supports that a technical limitation of the currently available diagnostic tools might be at play [12].

Our findings offer, for the first time, a possible explanation for the phenomenon of non-seroconversion, observed in a proportion of COVID-19 patients. Herein, we present evidence suggesting that delayed and/or absent antibody kinetics could probably be due to the prolonged and increased presence of viral constituents or even the virus in serum, saturating the antibodies, thus rendering their detection feasible only when antigenemia/viremia drops below a certain threshold (Figure 2B). Similar to the prolonged shedding seen in nasopharyngeal and gastrointestinal tract secretions, it is likely that such an event could take place in the blood stream, as well [27]. A probable source of antigenemia/viremia could be PANoptosis (pyroptosis, apoptosis, necroptosis), whereby cellular contents are released into the circulation [28]. A similar explanation has been proposed for the presence of viral RNA in the blood, reflecting a wash-out phenomenon from primary sites of infection [9,29].

The presence of viral RNAemia, detected by PCR assays, has beenexamined in several studies, especially in critical ill patients but the rate of detection is generally low and highly variable amongst them [8,9,10]. The different PCR protocols used (different primer sets, different target-gene amplification, etc.) and serum handling might explain such result heterogeneity. On the other hand, its low detection rate in the blood likely represents an underestimation as nucleic acids are inherently labile structures and thus, prone to degradation. Opposingly, detection of viral proteins in the bloodstream is an attractable target as they are more stable and degradation resistant; as such, coidentification of viral antigens and RNA is confirmed only in a subgroup of COVID-19 patients [30].

The take home message from the current effort is that antigenemia/viremia may affect SARS-CoV-2 antibody test results, possibly resulting in characterization of certain COVID-19 cases as non-responders [24]. In this context, such a phenomenon may lead to delay in or absence of seroconversion secondary to immune interference/competition due to immunocomplexes formation (Supplementary Figure S3) [31]. It is shown that the ratio between antigens and immunoglobulins, rather than the absolute values thereof, at a given timepoint during propagation of immune responses determines the result of the immunodiagnostic method (Figure 2B). As a result, such a confounding factor should be taken into consideration when interpreting test results. Moreover, it could serve as a partial explanation in discrepancies observed between various diagnostic tools monitoring antibody kinetics, as they could be variably affected by such a confounder. This variability is also demonstrated through head-to-head comparison of a lateral flow antibody test and our ‘in-house’ ELISA in the presented case (Figure 2B) [24,32]. Both assays, despite being subjected to the same masking effect imposed by antigenemia, have contradictory results (negative and positive, respectively) for the same sample tested. Specifically, in the case of lateral flow antibody test, it is likely that the antibody/antigen ratio exceeds assay’s free-antibody detection limit, not allowing adherence of antigen-unbound antibody to the strip, sufficient enough to produce a detectable signal (negative result).

Although the observed data come from prolonged follow-up (approximately 2 months) of a single case and may have limited generalizability, it should act as springboard for further investigation. We challenge the scientific community to examine the role of antigenemia as a confounding factor in immunodiagnostic assays. As such, immune response monitoring should be evaluated in longer intervals to better estimate population seroprevalence, thus designing tailored public health strategies (diagnostic algorithms). Such strategies, within the context of vaccine shortage, could potentially include vaccination prioritization of most vulnerable groups lacking antibody protection against SARS-CoV-2 re-infection [33].

## Figures and Tables

**Table 1:** Patient’s clinical information.

Demographic Information		
Age	19	
Sex	Male	
Weight	95 kg	
Height	175 cm	
BMI	31 kg/m^2^	
**Past Medical History**		
Underlying disease	Prediabetes	
Medication	Metformin	
Allergies	No	
Smoking history	Non-smoker	
Alcohol consumption	Socially	
**Family History**		
Father	Prediabetes	
Mother	Hypertension	
**History of Present illness**		
Onset of COVID-19 symptoms	12 October 2020 (Day 1)	
Symptoms	Fever	
Duration of symptoms	12 October 2020–14 October 2020 (Day 1–3; Fever up to 38.4 °C) 25 October 2020–29 October 2020 (Day 14–18; Fever up to 37.4 °C)	
PCR tests (nasopharyngeal swab specimens)	13 October 2020 (Day 2; Ct = 15) 27 October 2020 (Day 16; Ct = 30)	
Days of blood sampling	O.D. values	
	Antibody assay	Antigen Assay
Day 6	0.046 ± 0.005	0.313 ± 0.028
Day 11	0.066 ± 0.011	0.332 ± 0.010
Day 16	0.056 ± 0.007	0.325 ± 0.008
Day 20	0.064 ± 0.019	0.307 ± 0.023
Day 27	0.055 ± 0.004	0.277 ± 0.016
Day 55	0.167 ± 0.024	0.249 ± 0.032
Day 73	0.200 ± 0.009	0.233 ± 0.021

## Data Availability

All data presented in this manuscript have not been shared or presented in any meetings.

## Supplementary Materials

The following are available online at https://www.mdpi.com/article/10.3390/v13061143/s1, Figure S1: Anti-SARS-CoV-2 monoclonal antibody, Figure S2. RNA sequencing data (A) and associated genomic mutations (B) of isolated SARS-CoV-2 strain, Figure S3. Possible mechanistic explanation undermining masking of immune responses by antigenemia/viremia.
