# Supplementary material for: SARS-CoV-2 Antigenemia as a Confounding Factor in Immunodiagnostic Assays: A Case Study
Source: Viruses. 2021 Jun 14;13(6):1143. doi: 10.3390/v13061143 (PMC8232125; doi:10.3390/v13061143)
Supplement: Supplementary file 1 [file viruses-13-01143-s001.zip › viruses-1257540-supplementary.pdf]

viruses

MDPI

Supplementary Figures:

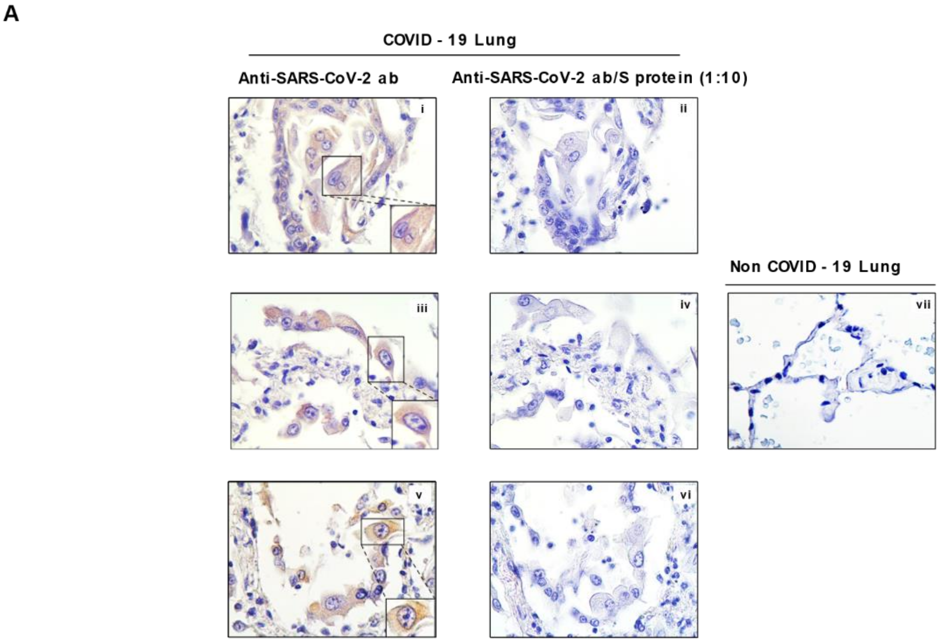

B

i

Double Antigen ELISA fot Antibody Detection

	COVID-19 Patients	Non COVID-19 Patients	Total
Positive Test Result	226	7	233
Negative Test Result	24	143	167
Total	250	150	400
	Sensitivity 90.5%	Specificity: 95%	

ii

Sandwich ELISA for Antigen Detection in Serum

	COVID-19 Patients	Non COVID-19 Patients	
Positive Test Result	26	1	27
Negative Test Result	2	71	73
Total	28	72	100
	Sensitivity: 93%	Specificity: 99%	

iii

Sandwich ELISA for Antigen Detection in Nasopharyngeal Swabs

	COVID-19 Patients	Non COVID-19 Patients	Total
Positive Test Result	44	1	45
Negative Test Result	2	123	125
Total	46	124	170
	Sensitivity 96.5%	Specificity: 99%	

Figure S1. Anti-SARS-CoV-2 monoclonal antibody (ab) immunohistochemical analysis (**A**) and performance characteristics of 'in-house' diagnostic assays (**B**). A. Immunohistochemical images from lung tissues of a positive COVID-19 patient (images i-vi) and a negative control (image vii). Cytoplasmic staining of Type II Pneumocytes is seen following incubation with secondary Anti-SARS-CoV-2 monoclonal antibody (479-G2) with (ii, iv, vi) and without (i, iii, v) competition with excess (one in ten dilution) viral Spike (S) protein. Upon competition, cytoplasmic signal is lost (ii, iv, vi). B. Performance

characteristics (sensitivity and specificity expressed in percentages) of 'in-house' assays: i. Double Antigen ELISA for antibody detection, ii. Sandwich ELISA for antigen detection in serum, and iii. Sandwich ELISA for antigen detection in nasopharyngeal swab specimens.

A

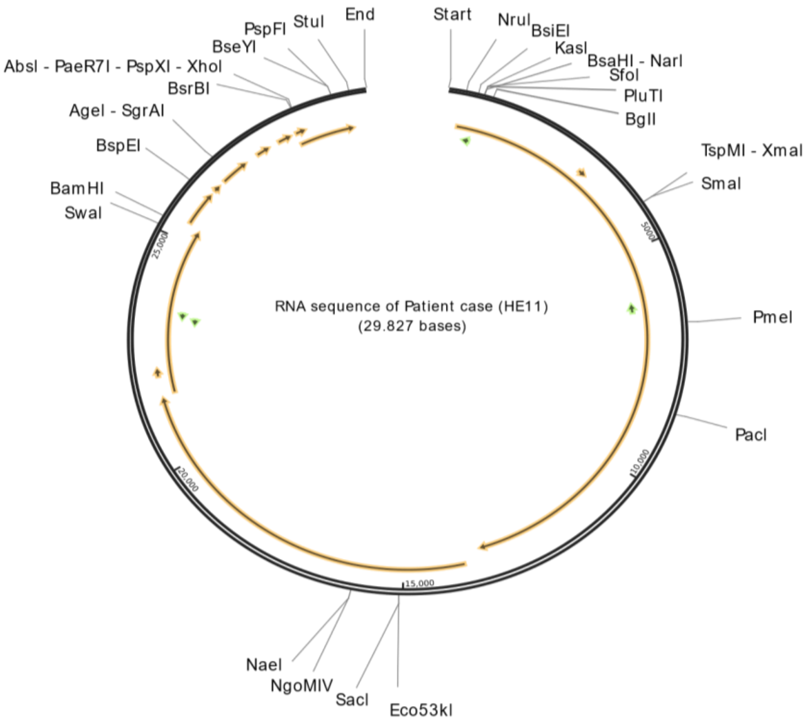

B

List of Mutations	Gene harboring substitutions
Q1842R	NSP3
V1897I	NSP3
V34F	NSP8
P323L	NSP12
D614G	Spike
A879S	Spike
M260K	NS3
T76I	N
R203K	N
G204R	N
D401Y	N

Figure S2. RNA sequencing data (**A**) and associated genomic mutations (**B**) of isolated SARS-CoV-2 strain. A. RNA sequencing of SARS-CoV-2 strain isolated from present case. Upon comparison against Wuhan-Hu-1 complete genome, 99% coverage similarity was observed (GISAID; Submission reference: EPI_ISL_856971). B. Table listing mutations and their corresponding gene locus within SARS-CoV-2 genome.

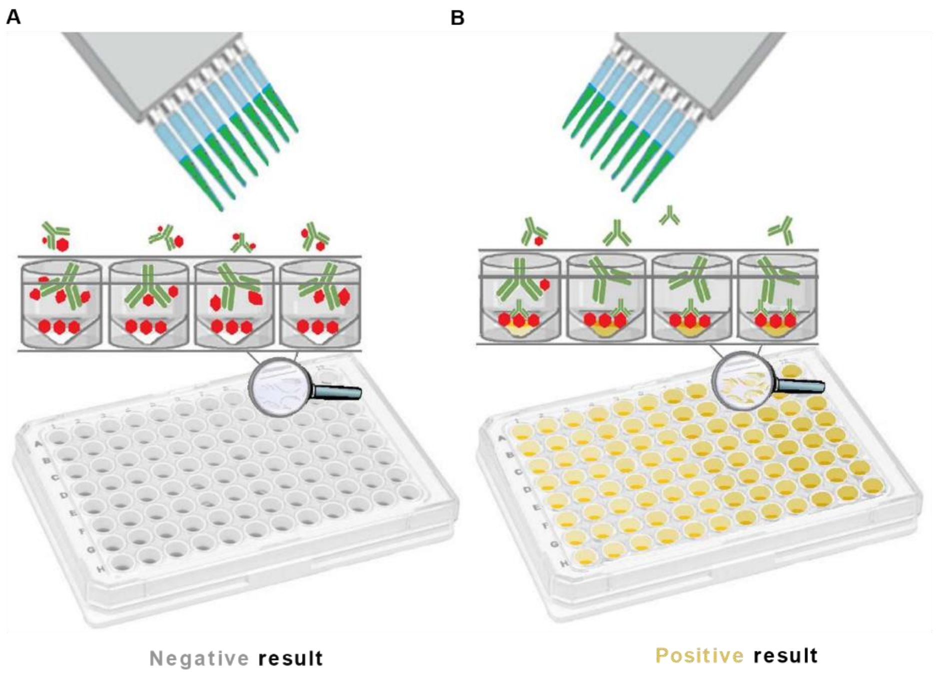

Figure S3. Possible mechanistic explanation undermining masking of immune responses by antigenemia/viremia. (**A**) A plausible explanation for interference may be that anti-SARS-CoV-2 antibodies present in the serum may directly cross react to viral antigens and/or virus also found in the serum. Subsequently, the association between anti-SARS-CoV-2 antibodies and viral antigens in the serum will inhibit the cross reaction of anti-SARS-CoV-2 antibodies with SARS-CoV-2 antigens immobilized on ELISA microplates (negative result). (**B**) We also suggest that when antigen/viral load decreases, this interference/competitive effect is alleviated, permitting serum anti-SARS-CoV-2 antibodies to cross react to immobilized antigen on the ELISA plate and hence being detected (positive result). Competitive ELISA is an established practice in immunoassays where the affinity between an antigen and antibody is tested. We propose that this effect may also occur during serological diagnostic tests for SARS-CoV-2, where antigenemia/viremia observed in COVID-19 will directly interfere with the immunoassay. Presumably, this may also extend to other infectious viral diseases where antigenemia/viremia has been observed.
